# Sequence-similar, structure-dissimilar protein pairs in the PDB

**DOI:** 10.1002/prot.21770

**Published:** 2007-11-14

**Authors:** Mickey Kosloff, Rachel Kolodny

**Affiliations:** 1 Department of Biochemistry and Molecular Biophysics, Center for Computational Biology and Bioinformatics, Columbia UniversityNew York, New York 10032; 1 Howard Hughes Medical Institute

**Keywords:** structure comparison, structure alignment, structural differences, nonredundant, structure prediction

## Abstract

It is often assumed that in the Protein Data Bank (PDB), two proteins with similar sequences will also have similar structures. Accordingly, it has proved useful to develop subsets of the PDB from which “redundant” structures have been removed, based on a sequence-based criterion for similarity. Similarly, when predicting protein structure using homology modeling, if a template structure for modeling a target sequence is selected by sequence alone, this implicitly assumes that all sequence-similar templates are equivalent. Here, we show that this assumption is often not correct and that standard approaches to create subsets of the PDB can lead to the loss of structurally and functionally important information. We have carried out sequence-based structural superpositions and geometry-based structural alignments of a large number of protein pairs to determine the extent to which sequence similarity ensures structural similarity. We find many examples where two proteins that are similar in sequence have structures that differ significantly from one another. The source of the structural differences usually has a functional basis. The number of such proteins pairs that are identified and the magnitude of the dissimilarity depend on the approach that is used to calculate the differences; in particular sequence-based structure superpositioning will identify a larger number of structurally dissimilar pairs than geometry-based structural alignments. When two sequences can be aligned in a statistically meaningful way, sequence-based structural superpositioning provides a meaningful measure of structural differences. This approach and geometry-based structure alignments reveal somewhat different information and one or the other might be preferable in a given application. Our results suggest that in some cases, notably homology modeling, the common use of nonredundant datasets, culled from the PDB based on sequence, may mask important structural and functional information. We have established a data base of sequence-similar, structurally dissimilar protein pairs that will help address this problem (http://luna.bioc.columbia.edu/rachel/seqsimstrdiff.htm).

## INTRODUCTION

It is often assumed that in the Protein Data Bank (PDB),^1^ all the structural representatives of a protein are similar, and more generally that two proteins with similar sequences will also have similar structures. Since the PDB includes many such pairs of structures, it has proved useful to develop subsets of the PDB from which “redundant” structures have been removed, based on a sequence-based criterion for similarity (e.g. Refs.^2^–^6^). These “non-redundant” subsets are often used in statistical and rule-based approaches to protein structure analysis and prediction. The implicit assumptions used in their construction is either that sequence-similar pairs in the PDB have insignificant structural differences or that if significant structural differences between such pairs do exist, the occurrence of this phenomenon is rare enough that it can be safely ignored. Similarly, when predicting protein structure using homology modeling, if a template structure for modeling a target sequence is selected by sequence alone, this implicitly assumes that all sequence-similar templates are equivalent.^7^ In particular, this assumption underlies most automated homology modeling servers. Here we investigate the validity of these assumptions.

Some time ago Chothia and Lesk^8^ observed that two structures with 50% (100%) sequence identity will align to ∼1 Å (0.6 Å) RMSD from each other. Sander and Schneider^9^ showed that two structures with more than 35 aligned residues and at least 40% sequence identity will generally structurally align to within 2.5 Å RMSD. Rost^10^ used a larger PDB to study the “twilight zone” of low-sequence identities and confirmed that sequence-similar proteins are expected to be structurally similar. Because these studies measured similarity between protein pairs, they focused on the common substructures and ignored dissimilar parts. Nonetheless, their results suggest that the similar-sequence implies similar-structure paradigm holds.

Of course, there are many well-known examples where proteins undergo significant conformational changes and in such cases the relationship between sequence and structural similarity may no longer be valid (for examples see Refs.^11^–^13^). The molecular motion database of Gerstein and co-workers,^12^–^15^ contains examples of proteins in the PDB with globally similar sequences and dissimilar structures. Most of the entries in this database are from a dataset built as a comprehensive sample of protein flexibility. The motions dataset contains over 3800 SCOP domain pairs sharing a fold, and with a pairwise RMSD that is two standard deviations higher than the average RMSD observed at a given percent identity.^16^,^17^ Recently, Gan *et al.* used structural alignment to compare a representative set of proteins selected from the PROSITE database of protein families and observed over 1700 pairs of structurally-dissimilar proteins in the PDB with sequence identities ≥20% and RMSD ≥ 2 Å.^18^ In these datasets, only a small minority of the structurally-dissimilar pairs have a sequence identity that is above 50% and very few of these have an RMSD ≥ 3 Å.

In this study, we further investigate the occurrence of protein pairs with similar-sequences and significant structure dissimilarity, focusing on pairs of proteins with high levels of sequence identity. In contrast to previous studies that used geometry-based structural alignment of protein pairs, our analysis is based on sequence-based structure superpositions, as we show that it better estimates structural differences in sequence-similar proteins. We find numerous protein pairs, of 50–100% sequence identity, that have dissimilar structures, as measured by RMSDs greater than 3 Å or 6 Å. A database of structure-dissimilar pairs is available online at http://luna.bioc.columbia.edu/rachel/seqsimstrdiff.htm. Our results suggest that when creating non-redundant subsets of the PDB or when selecting templates for homology modeling, two proteins or domains in the PDB should be judged as redundant only if both their sequences and structures are similar.

## RESULTS

### Structure alignment underestimates structural dissimilarity as compared to sequence-based structure superpositioning

It is useful to define the terms *alignment* and *superposition* as used in this work. An alignment of two proteins matches pairs of residues, one from each protein—the alignment refers to the set of these matched residues. *Superposition* refers to the process of superimposing, or overlaying, two protein structures in three dimensions. A *sequence-based superposition* is obtained by optimally superimposing all pairs of residues that are aligned by sequence alone (see Materials and Methods). In contrast, geometry-based superposition methods search for geometric similarities between two proteins while ignoring sequence information. Such programs align and superimpose structurally similar regions and assign gaps to regions that do not superimpose well. The commonly used term *structural alignment* refers to the coupled geometry-based superposition and the alignment it produces.

**Figure 1:**
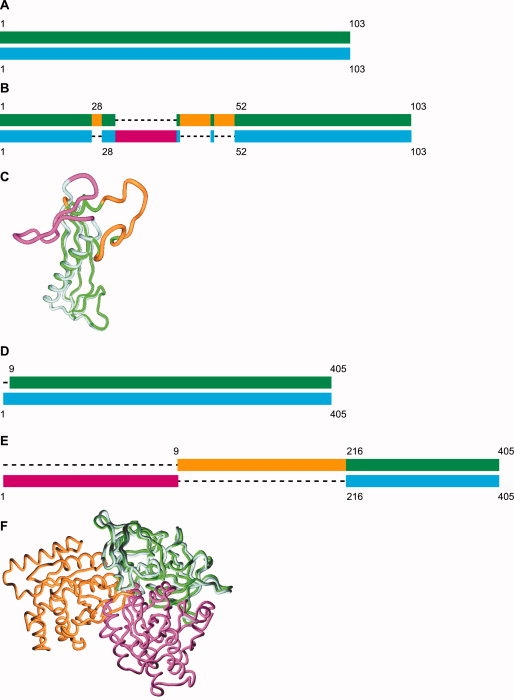
Two examples of how structure alignment can underestimate structural dissimilarity. (A, B) Schematic representation of the sequence alignment (**A**) versus structural alignment (**B**) of chain A versus chain D from PDB ID 1vr4. The two chains are 100% identical in sequence. The aligned parts are colored green (chain A) and cyan (chain D), while the unaligned parts are colored orange and magenta, respectively. The RMSD of all sequence-aligned residues is 7.1 Å, while that of the structurally-aligned residues is 1.4 Å. (**C**) Structural-alignment based superposition of chains A and D of 1vr4 (colored as in panel B). (D, E) Schematic representation of the sequence alignment (**D**) versus structural alignment (**E**) of two structures of the elongation factor Ef-Tu from Thermus aquaticus—PDB ID 1tui chain A (GDP bound) and 1eft (GTP bound). Inter-domain changes (hinge motion) cause structure alignment programs to align only one domain and ignore the rest of the protein. The aligned parts are colored green (1tui) and cyan (1eft), while the unaligned parts are colored orange and magenta, respectively. The RMSD of all sequence-aligned residues is 10.3 Å, while that of the structurally-aligned residues is 1.3 Å. Note that 1tuiA is not in our dataset because this structure was solved at a resolution of 2.7 Å. Homologs of 1tuiA from E.coli, with ∼70% sequence identity to 1tuiA and to 1eft are in our dataset. These orthologs (e.g. 1dg1G, 1d8tA) are similar in structure to 1tuiA and dissimilar to 1eft, with RMSDs > 10 Å to the latter structure. (**F**) Structural-alignment based superposition of 1tui chain A and 1eft (colored as in panel E).

The RMSD between two superimposed structures is usually measured only over those residues that are considered as aligned, that is, that are not assigned to gaps. The geometry-based alignment of two proteins that are similar in sequence and substantially different in structure will align fewer residues than will be aligned based on sequence. For example, Figure 1 shows two examples of how the RMSD obtained from a geometry-based structure alignment of two sequence-identical, or nearly identical chains, can be lower than that calculated by sequence-based structure superposition. In the first example (panels A–C) a local structurally-divergent region results in an RMSD of 7.13 Å when measured over all residues that are aligned by sequence. In contrast, the RMSD obtained from the structural alignment is much smaller (1.44 Å) since this RMSD is measured only over residues that occupy similar positions in space. In the second example (panels D–F), a hinge motion between domains causes the structure alignment programs to align only one domain and ignore the rest of the protein, resulting in an RMSD of 1.35 Å, which is significantly lower than that measured over all sequence-aligned residues that includes all residues in the full-length protein (10.28 Å).

**Figure 2:**
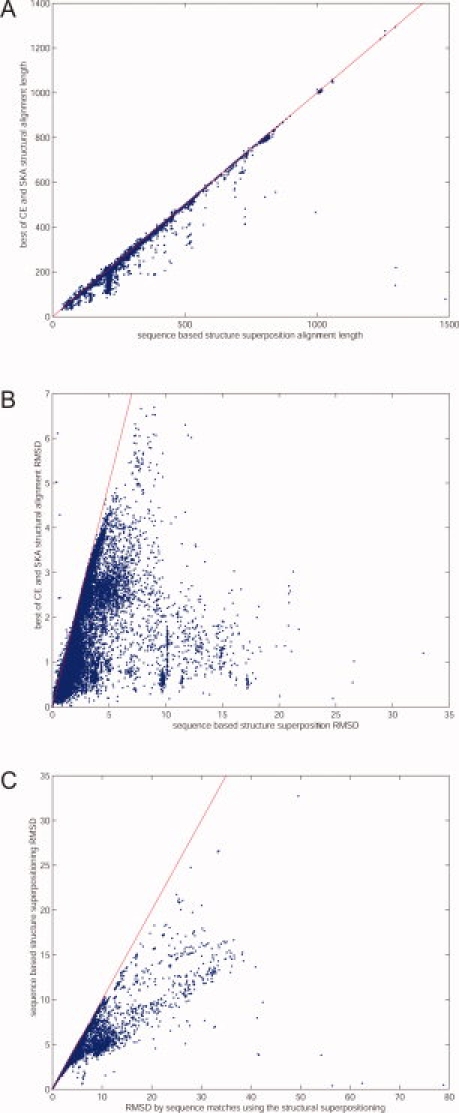
Comparison of sequence-based structural superpositioning and structural alignments. (**A**) sequence- and structure-alignment lengths. (**B**) RMSDs over these aligned sub-structures. Note the different scales of the x- and y-axis. (**C**) RMSDs of all sequence-aligned residue pairs using two different superpositions: on the x-axis using structural alignment superpositioning, and on the y-axis the sequence-based structural superpositioning. The aligned dataset is of protein pairs with sequence identity ≥70% (see Materials and Methods for details). [Color figure can be viewed in the online issue, which is available at www.interscience.wiley.com.]

Figure 2 compares the results of sequence-based structure superpositioning with geometry-based structure alignments obtained using the programs Ska^19^ and CE.^20^ Each data point shown in the figure corresponds to the best alignment obtained from one of the two programs (see Materials and Methods). The figure is based on a total of 110,068 protein pairs with sequence identities ≥70% (see Materials and Methods). As can be seen in Figure 2(A), structural alignment methods consistently align an equal number or fewer residues than sequence alignments. Figure 2(B) compares the RMSDs of the aligned sub-structures obtained from both approaches and further analysis of this data is presented in the Supplementary Material. In almost all cases, geometry-based structure alignments yield a lower RMSD than sequence-based RMSDs.

The situation is reversed when comparing alignments over the same set of residues. Figure 2(C) lists RMSDs calculated from either sequence or geometry-based superpositioning over the set of residues that are matched by the sequence alignments. That is, the RMSD is calculated for the same set of residues, including residues that are not aligned in the geometry-based alignments. In this case, the RMSD obtained from the geometry-based superposition is always larger than that obtained from the sequence-based superposition. This is expected since the structure alignment makes no attempt to align residues that are identified as equivalent in the sequence-based alignment. Thus, these residues are effectively ignored in the optimization procedure and the need to include them in the RMSD calculation will increase the value that is obtained.

### Protein pairs with similar sequences and significant structural differences

**Figure 3:**
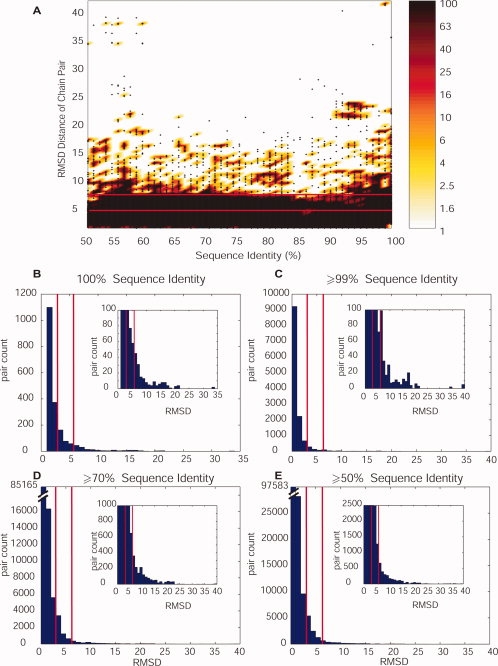
Abundance of sequence-similar and structurally-dissimilar pairs. (**A**) Sequence-based RMSD of all chain pairs in our data set versus their BLAST sequence identity; the color/gray scale codes the number of pairs in each area of the plot. (**B**–**E**) show the number of pairs of varying RMSDs and sequence identities ≥100, 99, 70, and 50%; the insets show the same histograms with a magnified y-axis scale. The data shown is the same as in (A), reorganized to quantify the abundance of different pairs with a given RMSD. Lines mark the 6 Å and 3 Å RMSD values. Notice that since we filter pairs with identical sequences and highly similar structures, there are no pairs with 100% sequence identity and less than 1 Å RMSD. [Color figure can be viewed in the online issue, which is available at www.interscience.wiley.com.]

Figure 3 shows the distribution of RMSD values obtained from sequence-based structure superpositioning for chain pairs of varying sequence identities. It is evident from the figure that there are many pairs of proteins that have high levels of sequence identity but that are structurally quite dissimilar. Table I lists the total number of pairs in 12 (overlapping) subsets defined by sequence identities ≥50, 70, 99, and 100% and RMSD ≥ 0 Å, 3 Å, 6 Å, showing many protein pairs with similar sequences and substantially different structures. For example, there are over 2600 (11,700) pairs with sequence identity greater or equal to 50% and RMSDs ≥ 6 Å (≥3 Å). Even for 100% sequence identities, there are 158 pairs with RMSD ≥ 6 Å. Note that had we based our analysis on geometry-based structure alignments, much fewer cases would have been detected.

**Table I:** Sequence-Similar, Structurally-Dissimilar Chain Pairs

	Total pairs^a^		
Sequence identity (%)	≥0 (Å)^b^	≥3 (Å)^c^	≥6 (Å)^c^
100	1,941	444	158
≥99	12,868	757	278
≥70	114,021	6,873	1,575
≥50	147,186	11,749	2,653

a Number of pairs after removing redundant structures from the PDB (see Materials and Methods).

b The total number of pairs for each of the four subsets.

c The total number of structurally-dissimilar pairs, restricted to RMSD **≥** 3 Å or 6 Å.

To relate our results to previous work, we used sequence-based structure superpositioning to analyze the “outlier set” in the molecular motions database.^16^,^17^ The majority of the domain pairs in the “outlier set” have sequence identities <50% and many contain NMR entries or structures with resolution worse than 2.5 Å. Only 742 pairs meet our criteria of RMSD ≥6 Å (3 Å), sequence identity ≥50% and resolution better than 2.5 Å. Similarly, the majority of the 1735 structurally dissimilar protein pairs reported by Gan *et al.* using representative probes^18^ do not meet our structure resolution, RMSD, and sequence identity criteria. Therefore, the vast majority of the sequence-similar structurally-dissimilar pairs that we report here have not been reported previously.

The complete list of chain pairs in our data set and the eight subsets of structurally-dissimilar chain-pairs (≥6 Å or ≥3 Å RMSD, sequence identity ≥50, 70, 99, and 100%) are available online (http://luna.bioc.columbia.edu/rachel/seqsimstrdiff.htm). Also available online is the sequence-based structural superposition of each pair.

We note that the protein pairs considered in this work cover a significant subset of SCOP families, superfamilies, and folds. Table II lists the number of unique SCOP v.1.69 classes, folds, superfamilies, and families counted in each of our subsets: pairs with sequence identities ≥50, 70, 99, and 100%, and RMSD ≥ 3 or 6 Å. The percent of all superfamilies that are found in the subset of pairs with sequence identity ≥50% (= 100%) ranges from 17.2% (7.9%) for RMSD ≥ 3 Å to 8.1% (3.1%) for RMSD ≥ 6 Å.

**Table II:** The Occurrence of Sequence-Similar, Structurally-Dissimilar Pairs In Different SCOP Classifications

	Number of SCOP v.1.69			
Pairs with sequence identity (%)	Classes (out of 9)	Folds (out of 945)	Super families (out of 1539)	Families (out of 2845)
Containing a structure from a pair with RMSD ≥ 6 Å				
100	8	44	48	54
≥99	8	51	56	63
≥70	9	99	111	129
≥50	9	112	125	150
Containing a structure from a pair with RMSD ≥ 3 Å				
100	8	104	122	143
≥99	8	124	149	179
≥70	9	190	238	306
≥50	9	209	265	351

### Inter versus intra-domain structural dissimilarities

Dissimilarities among structures of similar sequences can lie within (intra-) or across (inter-) domains. In the first case, sub-structures within a domain differ [e.g. Figs. 1(A–C)], while in the second there is a hinge motion between domains [e.g. Figs. 1(D–F)]. We can distinguish between these cases by comparing the RMSD over the full alignment with that of individually aligned domains. In the case of intra-domain dissimilarity both RMSD values will be the same, and large. For inter-domain dissimilarity the RMSD will be small when measured over individual domains separately [for example, in Fig. 1(D) the RMSD measured only over the superimposed cyan and green domains is small]. Here, we use the domain definitions of SCOP.

Table III lists the number and percentage of domain-pairs that have RMSD ≥ 6 Å or ≥ 3 Å for each of the four chain-pair subsets with chain RMSD ≥ 6 Å and both pairs classified in SCOP v.1.69. In each set, we consider all SCOP v.1.69 domain pairs that overlap more than 35 residues in their sequence alignment, and calculate the RMSD using only the aligned residues in the matched domains. In 60–80% of these structurally-different chain-pairs the RMSD measured over individual SCOP domain-pairs is also greater than 6 Å. Therefore, in the majority of the chain-pairs in this dataset, the structural dissimilarity is due to intra-domain differences.

**Table III:** Sequence-Similar, Structure-Dissimilar Chain Pairs Containing Structure-Dissimilar SCOP v.1.69 Domains (Intra-Domain Dissimilarity)

		Number of pairs containing at least one SCOP domain-pair with RMSD^b^	
Chain pairs with sequence identity (%)	Pairs with chain RMSD ≥ 6 Å^a^ (classified by SCOP)	≥6 (Å)	≥3 (Å)
100	148	106 (72%)	113 (76%)
≥99	259	208 (80%)	219 (85%)
≥70	1,338	987 (74%)	1,030 (77%)
≥50	2,289	1,422 (62%)	1,524 (67%)

a RMSD measured over all aligned residues in the chain pairs.

b Each chain is separated into SCOP domains and the RMSD is measured independently for each domain. When a chain contains multiple domains, the domain with the maximal *domain* RMSD **≥** 6 Å or 3 Å is counted. In parentheses are the percentages out of the number of chain pairs in each subset.

### Factors that lead to structural differences between sequence-identical proteins

**Figure 4:**
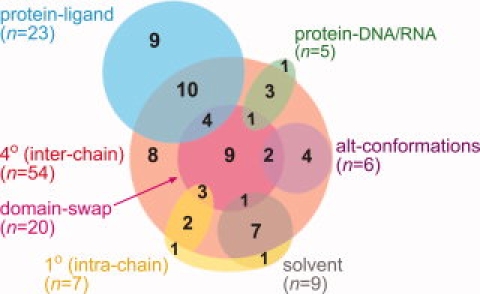
Causes for the marked structural dissimilarity between protein pairs with ≥99% sequence identity and RMSD ≥ 6 Å. The Venn diagram shows the distribution of causes for the structural dissimilarity within pairs. A detailed explanation of each category is given in the text. n refers to the number of occurrences of each cause, out of the 66 separate clusters examined.

Obviously, sequence differences are a major contributor to structural dissimilarity at lower levels of sequence identity. To identify the sources of structural differences between proteins that are essentially identical in sequence, we manually examined the set of 278 pairs in the ≥99% sequence identity and RMSD ≥ 6 Å subset of protein pairs. We clustered these pairs into 66 distinct clusters, based on their SCOP super-family classification and on their biological function, which was derived from the relevant literature. In almost all cases, the biological function dictates a conformational plasticity that results in two or more distinct structures. Figure 4 lists the distribution of causes that account for the structural differences observed for each pair in this subset. The full annotated subset is available online at (http://luna.bioc.columbia.edu/rachel/pairs_id99-100_rms6.html) and includes the full list of protein pairs and causes.

The causes of structural difference, ordered by frequency, are the following: (1) “Inter-chain (4° structure)”—different quaternary protein–protein interactions (including homomeric interactions). In the majority of cases this involves the presence of a protein chain, which interacts with the relevant chain in only one of the two structures in a pair. A minority of cases involve dissimilar interactions with similar binding partners (usually with an additional cause). “Domain-swap” is a sub-category of “inter-chain” interactions, where only one of the structures in a pair is domain-swapped.^21^,^22^ In rare instances both structures are domain-swapped, but with a different interface. (2) “Protein-ligand”—mostly a ligand-bound protein versus its apo form. Here, ligands are either small molecules, which are nonprotein/nonnucleic acid, or short (<15 residues) peptides. (3) “Solvent”—significant differences in the crystallization conditions (e.g. different pH or salt concentrations). (4) “Alt-conformations”—alternative crystallographic conformations of the same protein. Four of these cases are asymmetric homomers, for which “inter-chain” is an additional cause. One instance corresponds to the same protein crystallized in different space groups, and another corresponds to two alternative fits to the same crystallographic data. (5) “Intra-chain (1° structure)”—the presence/absence of part of a protein chain in one of the structures, a point mutation (combined with an additional cause), or in two instances, oxidized versus reduced intra-chain S—S bonds. (6) “Protein-DNA/RNA”—a DNA-bound protein versus its apo form. One instance involves a restriction enzyme (BamH) bound to specific versus non-specific DNA sequences.

**Figure 5:**
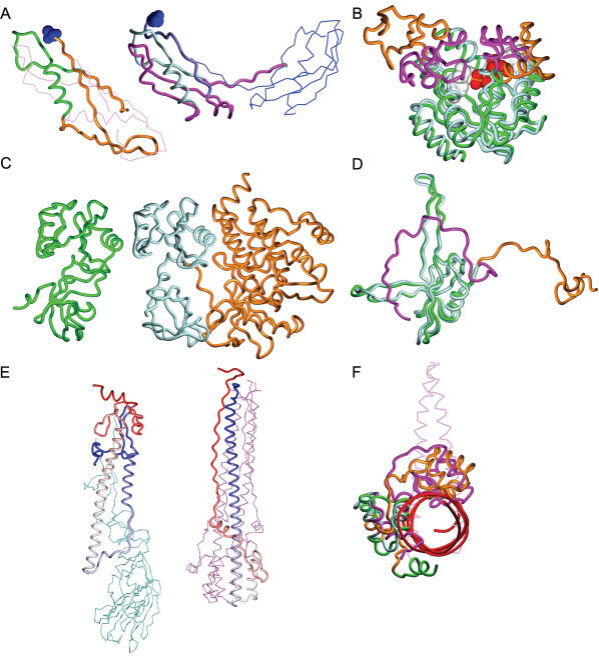
Examples of pairs with highly-similar sequences and structure dissimilarity that is related to biological function. (**A**) The bacterial protein TonB (1ihrB–1u07A, Inter-chain; Domain-swap; Intra-chain, RMSD of 20.4 Å, 100%): both compared structures are homodimers with a different domain-swapped interface, shown side by side for clarity. The structurally dissimilar regions are colored magenta (1u07A) and orange (1ihrB). 1u07 contains a 14 residue N-terminal stretch, depicted as a purple worm, which is absent in 1ihrB. The N-terminal residue of both compared chains is depicted in CPK model and the second monomer in each structure is depicted in Cα wire representation. (**B**) Adenylate kinase (1akeA–4akeA, Protein-ligand, RMSD of 7.1 Å, 100%): the ligand-bound form is superimposed on the apo form. The so called “lid” and “NMP” domains, which change conformation significantly upon ligand binding, are colored orange (1akeA, the apo form) and magenta (4akeA, the ligand-bound form). The ligand is depicted in CPK model. (**C**) The SH2-SH3 domains of cABL (2abl–1opkA, Intra-chain, RMSD of 9.5 Å, 95%): 1opk contains the cABL kinase domain (orange worm), which is absent in 2abl. This results in different SH2-SH3 domain-domain interaction (“inter-domain” differences). The two structures are displayed side-by-side for clarity. (**D**) The apo structure of the E.coli single-strand DNA-binding (SSB) protein (1qvcA–1qvcB, Inter-chain; Alt-conformations, RMSD of 20.7 Å, 100%): the two compared chains (out of four dissimilar chains in the homo-tetramer) are superimposed, and the variable C-terminus is colored orange (chain A) and magenta (chain B). (**E**) Influenza haemagglutinin (2viuB–1qu1F, Inter-chain; Solvent, RMSD of 39.8 Å, 94%): The two structures are displayed side-by-side for clarity and the two compared chains are colored in a gradient from blue (N-terminal) through white to red (C-terminal). The additional interacting chains are depicted in Cα wire representation. (**F**) The c-Myb transcription factor (1gv2A–1h89C, Inter-chain; Protein-DNA, RMSD of 7.1 Å, 100%): the apo form is superimposed on the DNA bound form, which also includes two chains of the C/EBPβ enhancer protein, depicted in Cα wire representation. The DNA backbone is depicted in red worm and the structurally variable regions are colored magenta (1gv2A) and orange (1h89C). In parenthesis for each example are the two protein chains, designated by their PDB id and chain ID, the causes for the structural differences between the two chains, the sequence-based superpositioning RMSD and the coverage (percentage of the alignment length from the length of the shorter chain). The compared chains are depicted as backbone worms. Unless stated otherwise, the first chain in each pair is colored green and the second chain cyan.

Figure 5 presents selected examples of functional significance that is related to the structural differences between high sequence identity pairs. These include: (a) The bacterial protein TonB (CASP6 target T0240), where the considerable structural difference (20.4 Å RMSD) is a result of intra-protein differences (one structure contains a 14 residue N-terminal stretch, which is absent in the other structure) and a different “inter-chain” quaternary structure, which in this case involves two disparate modes of domain-swapping within each homodimer. Biochemical evidence suggests that in additional to these two conformations, other conformations also exist.^23^ This inherent structural plasticity is thought to be central in TonB's function as a transport mediator.^23^,^24^ (b) The apo versus ligand-bound forms of adenylate kinase. The so called “lid” and “NMP” sub-domains change conformation upon ligand binding as part of the catalytic cycle of this enzyme,^25^ resulting in a 7.1 Å RMSD. (c) The SH2-SH3 domains of the cABL tyrosine kinase, with or without the C-terminal kinase domain. The presence of the kinase domain in one structure locks the SH2 domain in a specific conformation in relation to the SH3 domain,^26^ resulting in an RMSD of 9.5 Å to the second structure, which lacks the kinase domain.^27^ These two crystallographic snapshots are representative of a much wider array of possible conformations of cABL.^28^,^29^ (d) Alternative conformations of the monomers in the apo form of the E.coli single-strand DNA-binding (SSB) protein. Each C-terminus of the four chains in this homo-tetramer, which belongs to the nucleic-acid binding OB-fold superfamily, adopts a different conformation. This conformational plasticity is consistent with the significant conformational changes and refolding events that have been generally associated with the function of nucleic-acid binding by OB-fold proteins.^30^ (e) Influenza haemagglutinin, a text book example of functional conformational change,^31^ where different pH (“solvent”) and differing “inter-chain” interactions result in the largest RMSD difference (39.8 Å) in this high identity subset. (f) The apo form of the transcription factor and proto-oncogene c-Myb versus its DNA bound form. The latter structure also contains an additional transcription factor, C/EBPb, which interacts with c-Myb and the bound DNA.^32^

### Examples of sequence-similar, structure-dissimilar templates for homology modeling

The frequent occurrence of sequence-similar, structure-dissimilar proteins in the PDB, which we observe, poses a unique challenge to homology modeling. In particular, we are not aware of an automatic homology modeling server that returns more than one alternative “best” model, if there is more than one sequence-equivalent, but structural dissimilar, template in the PDB. We illustrate how our database can identify such templates with two relatively “easy” examples of homology modeling.

(1) As shown in Figure 5(A), TonB from *E.coli* has been crystallized in two alternative homodimeric forms. If, for example, we want to model a C-terminal part of TonB from *Enterobacter aerogenes* (residues 171–240 of Uniprot entry TONB_ENTAE), searching with this sequence for homologs identifies the *E.coli* structures (1ihrB and 1u07A), both aligning with 75% sequence identity and no gaps to the *E. aerogenes* sequence. Searching our database for either structure identifies the 1ihrB-1u07A pair as having identical sequences and a sequence-based superpositioning RMSD of 20.4 Å. Therefore, these two templates should be treated as non-redundant, and the user modeling the *E. aerogenes* sequence needs to decide between the two alternative templates based on biological or functional criteria. Similarly, an automated prediction server should return both alternative models. Indeed, the assessors in the CASP6 experiment noted that target T0240 (TonB from *E.coli*) was a difficult target for prediction and an “odd case” that “fooled” the automatic prediction servers and thus had to be removed from some of the assessments.^33^

(2) In a second example we consider modeling the SH2-SH3 domains of the ABL kinase from *Drosophila melanogaster* (residues 187–346 of Uniprot entry ABL_DROME). Searching for homologs of this query, we find the vertebrate structures (2abl and 1opkA) that align with 74% sequence identity to the *D. melanogaster* sequence with almost no gaps. Figure 5(C) shows these templates, where the SH2-SH3 domains of the cABL kinase have different conformations, depending on the presence or absence of the kinase domain. Our database shows that the 2abl-1opk chain pair show 100% sequence identity and a sequence-based superpositioning RMSD of 9.5 Å. The graphical representation of the 2abl-1opkA alignment (Fig. S2 of the Supplementary Material) illustrates that the C-terminal kinase domain is present only in 1opk. Here, choosing one of these templates over the other to model the *D. melanogaster* sequence must be based on the biological context of the model.^31^ Interestingly, searching for 1opk in our RMSD ≥ 3 Å subset identifies two more structure-dissimilar homologs, 1opjB and 1fpuB, that are essentially identical in sequence to the C-terminal domain of 1opk. These are structures of the kinase domain of cABL (i.e., they do not overlap with 2abl, see Fig. S2 in the Supplementary Material), which show an intra-domain dissimilarity to 1opk. The sequence-based superpositioning RMSD of 1opjB and 1fpuB to 1opkA is 5.1 and 3.7 Å, respectively. The reader is referred to Nagar *et al.* for a detailed discussion of the biological significance and cause of the structural differences in the different domains of cABL.^31^

## DISCUSSION

In this article we report the existence of a significant number of sequence-similar structurally-dissimilar pairs of proteins in the PDB. Although numerous, these pairs are a minority in our dataset, and by extension in the PDB. The structural dissimilarities range from global rearrangements through inter-domain motion to relatively local structural differences (see also Supplementary Material). The majority of the cases correspond to intra-domain differences. Also, the range of SCOP classifications for the pairs of proteins that we find shows that this phenomenon is found in a wide range of biological families and structural folds.

Many of the pairs of proteins that we identify would not have been found with geometry-based structural alignment programs. As discussed earlier, such programs search for common sub-structures between two proteins, while removing dissimilar parts from the resulting alignment. Thus they will underestimate true geometric differences between structures. In contrast, had we used the results of the geometric superpositions to measure dissimilarities over regions that are well-aligned in sequence, we would have found even more cases of structurally dissimilar pairs. In this regard, it is important to emphasize here that since we are only considering pairs of proteins with high sequence identity (≥50%) and low E-values, the sequence alignments are quite reliable and hence sequence-based structure superpositioning provides a meaningful measure of structural dissimilarities. Furthermore, these high sequence identity alignments typically cover most of the aligned sequences: in the set of ≥70% sequence identity, more than 90% of the residues in both proteins are aligned in more than 95% of the pairs.

Interestingly, the vast majority of the sequence-similar structurally-dissimilar pairs reported here were not identified in the studies of Gerstein and co-workers or in the results of Gan *et al.*^17^,^18^ The apparent discrepancy results from a combination of three factors: (1) previous studies used geometry-based structural alignment to calculate RMSDs. (2) The majority of the pairs reported here were not in the PDB five years ago, when other databases were built (data not shown). (3) To reduce the computational cost associated with large-scale structure alignment, previous studies used a representative set of structures, reduced according to SCOP or PROSITE classification. However, our results suggest that many sequence-similar pairs will be overlooked when considering such a reduced set.

Our estimate of the number of sequence-similar structurally-dissimilar protein pairs in the PDB is conservative because: (1) NMR structures were removed from our dataset. (2) The resolution and length thresholds remove many known examples of conformational changes (see examples in Refs.^12^,^13^). (3) We only compare single PDB chains and ignore relative structure changes in a complex of multiple chains within PDB entries.^34^ (4) Using global RMSD as a measure of dissimilarity understates relatively local changes in larger proteins.

As is well known, lower sequence identity between pairs contributes to structural differences.^35^ This effect is eliminated when focusing on a very high identity/dissimilar structures subset. Our classification of the environmental causes in this subset shows that distinct inter-chain (protein–protein) interactions account for more than half of the dissimilarities and differing protein-ligand interactions for more than one third. This reflects the known fact that binding is often associated with significant conformational changes. As expected, in all of the cases we surveyed that had a known biological function, the conformational plasticity leading to multiple structural states was dictated by that function. It should be noted that the biological function that underlies conformational plasticity is not necessarily the direct cause of the structural differences: for example, a protein that is flexible because of its DNA binding function can adopt two different conformations, even in the absence of bound DNA.

From one perspective, the results of this study are not surprising. The fact that single proteins can exist in more than one conformation is well-known and thus it is expected that some pairs of proteins that are closely related in sequence will have significantly different structures. Disordered proteins are an even more extreme example of structural plasticity.^36^–^38^ However, we believe that the frequency of this phenomenon in the PDB comes as a surprise. It is large enough to suggest that culled databases that do not take structural plasticity into account may mask important information that can be used, for example, in homology model building. This is particularly relevant to automated structure prediction servers that generally provide a single model as their top answer and usually rely on non-redundant representations of the PDB and to the assessment of structure prediction methods, as in the CASP experiments.^7^ The database we have developed as a result of this study (http://luna.bioc.columbia.edu/rachel/seqsimstrdiff.htm) may prove useful in this regard.

Finally, the different results obtained from different alignment protocols raise issues about the meaning of structural alignments. Since geometry-based alignments search for common substructures, they can identify evolutionary related regions of two proteins that do not have a significant sequence similarity. However, when two sequences can be aligned in a statistically meaningful way, the identification of remote evolutionary relationships is not an issue. In this case, sequence-based structural superpositioning provides a meaningful measure of structural differences and of the extent of conformational change that a group of closely related proteins may be expected to undergo. In such cases, geometry-based structure alignments are only useful as a means of identifying common regions between two alternative conformations. Clearly the two approaches to superpositioning reveal different information and it may be useful to use one or both in different applications.

## MATERIALS AND METHODS

### Data set of protein chains

The dataset used here includes all protein chains from the April 2005 PDB, that are longer than 35 residues, and whose structures were determined to resolution 2.5 Å or better using X-ray crystallography; 38,449 chains from 19,295 proteins satisfy these criteria. Chains with sequence identity of 100% and RMSD lower than 1 Å over their corresponding Cα atoms are defined as redundant. For structures with a resolution of 2.5 Å or better, the Cα RMSD due to the experimental error is well below this threshold.^39^–^41^ In every set of redundant chains, the chain with better resolution was kept. In case of several redundant chains with identical resolution, the longest was kept. The final data set contains 13,193 chains from 9906 protein structures.

### Data set of protein chain pairs

The sequences of all chain pairs in the above data set were aligned with BLAST utility bl2seq (version 2.2.10).^42^,^43^ Alignments that had: (1) sequence identity greater or equal to 50%, (2) E-value better than 0.001, and (3) at least 35 matched residues, were selected, resulting in 147,186 pairs. Using more stringent E-value cutoffs up to 10^−10^ and increasing the alignment length cutoffs up to 70 matched residues had a negligible effect on the size of the dataset (data not shown). The sequences were extracted from the PDB coordinates (rather than from the SEQRES fields) and chemically modified residues were translated to standard residues as in ASTRAL.^44^ When creating this data set, pairs were filtered by masking low-complexity sub-sequences in the aligned pairs (BLAST filter parameter turned “on”), and then recalculating the correct sequence identity without masking low-complexity sub-sequences (filter parameter turned “off”).

### Sequence alignment and sequence-based structural superpositioning

As the focus here is on protein pairs that have well-aligned sequences, the set of matching residues can be extracted from their sequence alignment. Each protein pair in the data set was aligned, recording the E-value, BLAST score, sequence identity, sequence similarity, and alignment length. The matching residues were optimally superimposed and the RMSD was calculated using this superposition. Formally, a rotation and translation of one of the chains with respect to the other was calculated, so that it (globally) minimizes the RMSD of the Cα atoms of the sequence-aligned residues.^45^ This method is denoted sequence-based structure superpositioning. An implementation of sequence-based structure superpositioning is available within Vistal (http://luna.bioc.columbia.edu/∼kolodny/software.html).

The chain pairs were separated into 12 (overlapping) sets based on their level of sequence identity (≥50, 70, 99, and 100%) and structural similarity (RMSD greater than 0, 3, and 6 Å).

### Geometry-based structure alignment

Structural superpositions were carried out with Ska,^19^ and CE,^20^ and the corresponding alignment lengths and RMSDs were recorded. Ska and CE were combined into one alignment method by selecting the alignment with the lowest SAS score (SAS = RMS * 100/(number_matched_residues)).^46^ For comparing structure alignment with sequence-based superpositions we focus on cases that are expected to align similar regions using both approaches, restricting the analysis to the 110,068 chain pairs with sequence identities greater or equal to 70%, and with at least 90% of the residues in both chains aligned.

### Incorporating SCOP domain assignments into the data set

The SCOP v.1.69^47^ domain classification was used to assess if the structural dissimilarities are within domains (intra-domain), or if are they mostly due to inter-domain differences (i.e., rigid body movement of one domain relative to another domain in the same chain). For each SCOP-classified sequence-aligned pair, each structure was separated into SCOP domains and RMSDs were calculated independently for each of the sequence-aligned domain pairs, recording the maximum RMSD among the pairs of domains. We also count the different SCOP classifications of the aligned domains to gauge the diversity of the pairs in the sets. As SCOP does not classify all PDB entries, we verified that for all subsets, more than 96% of the chains and more than 85% of the chain-pairs are classified in SCOP v.1.69.
